# Stachydrine prevents LPS‐induced bone loss by inhibiting osteoclastogenesis via NF‐κB and Akt signalling

**DOI:** 10.1111/jcmm.14551

**Published:** 2019-07-21

**Authors:** Jiahong Meng, Chenhe Zhou, Wenkan Zhang, Wei Wang, Bin He, Bin Hu, Guangyao Jiang, Yangxin Wang, Jianqiao Hong, Sihao Li, Jiamin He, Shigui Yan, Weiqi Yan

**Affiliations:** ^1^ Department of Orthopedic Surgery, The Second Affiliated Hospital Zhejiang University School of Medicine Hangzhou China; ^2^ Orthopedic Research Institute of Zhejiang University Hangzhou China

**Keywords:** inflammatory osteolysis, NFATc1, NF‐κB, osteoclasts

## Abstract

Osteoclast overactivation‐induced imbalance in bone remodelling leads to pathological bone destruction, which is a characteristic of many osteolytic diseases such as rheumatoid arthritis, osteoporosis, periprosthetic osteolysis and periodontitis. Natural compounds that suppress osteoclast formation and function have therapeutic potential for treating these diseases. Stachydrine (STA) is a bioactive alkaloid isolated from *Leonurus heterophyllus* Sweet and possesses antioxidant, anti‐inflammatory, anticancer and cardioprotective properties. However, its effects on osteoclast formation and function have been rarely described. In the present study, we found that STA suppressed receptor activator of nuclear factor‐κB (NF‐κB) ligand (RANKL)‐induced osteoclast formation and bone resorption, and reduced osteoclast‐related gene expression in vitro. Mechanistically, STA inhibited RANKL‐induced activation of NF‐κB and Akt signalling, thus suppressing nuclear factor of activated T cells c1 induction and nuclear translocation. In addition, STA alleviated bone loss and reduced osteoclast number in a murine model of LPS‐induced inflammatory bone loss. STA also inhibited the activities of NF‐κB and NFATc1 in vivo. Together, these results suggest that STA effectively inhibits osteoclastogenesis both in vitro and in vivo and therefore is a potential option for treating osteoclast‐related diseases.

## 1 INTRODUCTION

Bone remodelling is a predominant metabolic process regulating bone structure and function and involves a balance between osteoclast‐derived bone resorption and osteoblast‐derived bone formation.^1^, ^2^ A disruption of this balance under pathological conditions aberrantly activates osteoclast activity, leading the dominance of bone resorption over bone formation and eventually bone destruction, as observed in chronic inflammatory diseases such as rheumatoid arthritis, osteoporosis, periprosthetic osteolysis and periodontitis.^3^, ^4^, ^5^ Therefore, strategies that modulate aberrant osteoclast activity have the potential to prevent inflammatory bone loss.

Osteoclasts, which are derived from hematopoietic monocyte/macrophage precursors, are multinucleated giant cells that are mainly involved in bone resorption. Macrophage colony‐stimulating factor (M‐CSF) and receptor activator for nuclear factor‐kappa B (NF‐κB) ligand (RANKL) are the key cytokines involved in osteoclast differentiation. The binding of RANKL to its receptor RANK promotes the recruitment of adaptor molecules called tumour necrosis factor receptor‐associated factors (TRAFs), which activate downstream signalling pathways, including NF‐κB and mitogen‐activated protein kinase (MAPK) pathways.^6^, ^7^, ^8^ This leads to the activation and accumulation of two pivotal transcription factors involved in osteoclast differentiation, namely, c‐Fos and nuclear factor of activated T cells c1 (NFATc1).^9^, ^10^ These factors induce the expression of osteoclast‐specific genes, including tartrate‐resistant acid phosphatase (TRAP), dendritic cell‐specific transmembrane protein (DC‐STAMP), cathepsin K (CTSK) and calcitonin receptor (CTR), leading to the formation of mature osteoclasts.^11^, ^12^ Subsequently, mature osteoclasts undergo polarization and structural changes to produce a tight sealing zone. Secretion of acids and proteolytic enzymes into this sealing zone results in the dissolution and degradation of the underlying bone.^13^, ^14^

Several recent studies have focused on natural compounds because of their pharmacological activity against human diseases. Over several years, our group has screened natural compounds for treating osteoclast‐related diseases.^13^, ^14^, ^15^, ^16^ Stachydrine (STA), a bioactive constituent extracted from a medicinal herb *Leonurus heterophyllus* Sweet, has extensive pharmacological properties, including antioxidant, anti‐inflammatory, anticancer and cardioprotective properties.^17^, ^18^, ^19^, ^20^ Recent studies have reported that STA inhibits NF‐κB and MAPK pathways in different cell types.^20^, ^21^, ^22^ As the NF‐κB and MAPK pathways are crucial for osteoclastogenesis and because STA exerts inhibitory effects on these pathways, we hypothesized that STA may represent a novel candidate for treating osteoclast‐related diseases by inducing the targeted suppression of osteoclastogenesis.

In the present study, we first investigated the effects of STA on RANKL‐induced osteoclast formation and bone resorption. Second, we assessed the effects of STA on crucial signalling events in RANKL‐induced osteoclastogenesis to determine its molecular target. Third, we explored the therapeutic potential of STA in a murine model of LPS‐induced inflammatory bone loss. Thus, our study determined the effect of STA on osteoclastogenesis and inflammatory bone loss and elucidated mechanisms underlying its mechanism of action.

## 2 MATERIALS AND METHODS

### 2.1 Reagents

Stachydrine (purity, ≥98%) and SC79 (purity, ≥97%) were purchased from Selleck Chemicals. Alpha modification of Eagle's medium (α‐MEM), penicillin/streptomycin and foetal bovine serum (FBS) were obtained from Gibco‐BRL. Recombinant mouse M‐CSF and RANKL were purchased from R&D Systems. Primary antibodies against TAK1 (#5206), phosphorylated TAK1 (p‐TAK1) (#4508), IκBα (#4814), phosphorylated IκBα (p‐IκBα) (#2859), p65 (#8242), phosphorylated p65 (p‐p65) (#3033), IκB kinase β (IKKβ) (#8943), phosphorylated IKKα/β (p‐IKKα/β) (#2697), p38 (#9212), phosphorylated p38 (p‐p38) (#4511), ERK (#4695), phosphorylated ERK (p‐ERK) (#4370), JNK (#9252), phosphorylated JNK (p‐JNK) (#4668), phosphorylated Akt (p‐Akt) (#2965), Akt (#4685), phosphorylated GSK3β (p‐GSK3β) (#9323), GSK3β (#9315), NFATc1 (#8032), PCNA (#13110) and β‐tubulin (#2146) were obtained from Cell Signaling Technology. Primary antibodies against c‐Fos (ab208942), NFATc1 (ab2796) and CTSK (ab37259) were obtained from Abcam. Primary antibody against TRAP (sc‐376875) was obtained from Santa Cruz Biotechnology and against active p65 (MAB3026) was obtained from Millipore. Cell counting kit‐8 (CCK‐8) was obtained from Dojindo Molecular Technology. TRAP staining kit, DMSO, SC‐514 and other reagents were obtained from Sigma‐Aldrich, unless otherwise indicated.

### 2.2 In vitro bone marrow‐derived macrophage isolation and osteoclast differentiation

Bone marrow cells were obtained from the long bones of 8‐week‐old male C57BL/6 mice, as described previously,^23^ and were differentiated into bone marrow‐derived macrophages (BMMs) in α‐MEM supplemented with 10% FBS, 100 U/mL penicillin, 100 µg/mL streptomycin and 25 ng/mL M‐CSF at 37°C for 5 days in a humidified incubator with 5% CO_2_. Next, BMMs were seeded into 48‐well plates (density, 1 × 10^4^ cells/well) in triplicate and were treated with various concentrations of STA (0, 12.5, 25, 50, 100 or 200 μmol/L) in the presence of 25 ng/mL M‐CSF and 50 ng/mL RANKL. Culture medium was replaced every 2 days. After culturing for 5 days, the cells were fixed with 4% paraformaldehyde (PFA) and stained for TRAP according to the manufacturer's protocol. TRAP‐positive multinucleated cells (nuclei number, ≥3) were counted using a light microscope (BX51; Olympus).

### 2.3 Cell viability assay

The potential cytotoxic effects of STA on BMMs were determined by performing CCK‐8 assay. BMMs were seeded in 96‐well plates (density, 5 × 10^3^ cells/well) in the presence of 25 ng/mL M‐CSF for 24 hours. The cells were then treated with different concentrations of STA (0‐800 µmol/L) for 48 or 96 hours. Next, 10 μL CCK‐8 buffer was added to each well, and the plates were incubated at 37°C for 2 hours. Absorbance was measured using ELX800 absorbance microplate reader (BioTek) at a wavelength of 450 nm (reference wavelength, 650 nm).

### 2.4 F‐actin ring immunofluorescence assay and resorption pit assay

To observe F‐actin ring formation, BMMs were cultured in the presence of 25 ng/mL M‐CSF and 50 ng/mL RANKL for 4 days. Next, an equal number of mature osteoclasts were seeded on an Osteo Assay Surface Multiple Well Plate (Corning) coated with hydroxyapatite. After culturing overnight to promote adhesion, the cells were treated with 0, 25, 50 and 100 µmol/L STA for 2 days. Next, the cells were fixed with 4% PFA for 20 minutes and were permeabilized using 0.5% Triton X‐100 for 20 minutes. The cells were then washed three times with PBS and were stained with rhodamine‐conjugated phalloidin (dilution, 1:200; Invitrogen Life Technologies) diluted in 1% bovine serum albumin (BSA) for 30 minutes. Images of F‐actin rings were captured using a fluorescence microscope (EU5888; Leica) and were analysed using ImageJ software (National Institutes of Health). To observe resorption pits, the cells adhering to the plates were removed by incubating with 5% NaClO for 10 minutes. Resorption pits were photographed using a light microscope (Olympus), and their areas were analysed using the ImageJ software.

### 2.5 Quantitative PCR

Total RNA was isolated from the cultured cells by using RNeasy Mini Kit (Qiagen) and was reverse transcribed to cDNA by using PrimeScript RT Master Mix (TaKaRa Biotechnology), according to the manufacturers’ protocols. Quantitative PCR (qPCR) was performed using ABI 7500 Sequencing Detection System (Applied Biosystems) with SYBR Premix Ex Taq Kit (TaKaRa Biotechnology). Each reaction was performed using 10 µL SYBR Premix, 1 µg cDNA and different primer sets for 40 cycles with the following conditions: denaturation at 95°C for 5 seconds, annealing at 60°C for 20 seconds and extension at 72°C for 20 seconds. GAPDH was used as a housekeeping gene. Mouse primer sequences used for performing qPCR in the present study are shown in Table 1.

**Table 1:** Primers used for quantitative PCR

Gene	Forward (F) and reverse (R) primer sequence (5′–3′)
GAPDH	F: ACCCAGAAGACTGTGGATGG
	R: CACATTGGGGGTAGGAACAC
CTSK	F: CTTCCAATACGTGCAGCAGA
	R: TCTTCAGGGCTTTCTCGTTC
TRAP	F: CTGGAGTGCACGATGCCAGCGACA
	R: TCCGTGCTCGGCGATGGACCAGA
DC‐STAMP	F: AAAACCCTTGGGCTGTTCTT
	R: AATCATGGACGACTCCTTGG
c‐Fos	F: CCAGTCAAGAGCATCAGCAA
	R: AAGTAGTGCAGCCCGGAGTA
NFATc1	F: CCGTTGCTTCCAGAAAATAACA
	R: TGTGGGATGTGAACTCGGAA
CTR	F: TGGTTGAGGTTGTGCCCA
	R: CTCGTGGGTTTGCCTCATC

### 2.6 Western blotting analysis

To determine the main signalling pathways targeted by STA, BMMs were seeded in six‐well plates (density, 8 × 10^5^ cells/well) with complete α‐MEM supplemented with 25 ng/mL M‐CSF and allowed to adhere overnight. After treatment with DMSO or 100 μmol/L STA for 4 hours, the cells were stimulated with 50 ng/mL RANKL for 0, 5, 10, 20, 30 or 60 minutes. To examine the effects of STA on the expression of osteoclast‐related markers, BMMs were seeded in six‐well plates (density, 1 × 10^5^ cells/well) and were stimulated with 25 ng/mL M‐CSF and 50 ng/mL RANKL in the presence or absence of 100 μmol/L STA for 0, 1, 3 or 5 days. Next, the cells were washed twice with PBS, lysed using a radioimmunoprecipitation assay (RIPA) lysis buffer (Sigma‐Aldrich) on ice for 30 minutes. Each protein lysate containing 30 μg protein was analysed on 8%‐12% sodium dodecyl sulphate‐polyacrylamide gel electrophoresis (SDS‐PAGE), and resolved proteins were transferred onto polyvinylidene difluoride (PVDF) membranes (Millipore). The membranes were blocked with 5% skimmed milk for 1 hours, followed by overnight incubation at 4°C with the primary antibodies. After three washes, the membranes were incubated with appropriate horseradish peroxidase‐conjugated secondary antibodies at 4°C for 2 hours. Signals were detected using an electrochemical luminescence reagent (Millipore) and were visualized using XRS chemiluminescence detection system (Bio‐Rad).

### 2.7 Analysis of NFATc1 nuclear translocation

Extracts for determining NFATc1 nuclear translocation were prepared from BMMs stimulated with 25 ng/mL M‐CSF and 50 ng/mL RANKL in the presence or absence of 100 μmol/L STA for 0, 2 or 4 days. Cytoplasmic and nuclear proteins were extracted using Nuclear and Cytoplasmic Protein Extraction Kit (Beyotime), according to the manufacturer's protocol. Western blotting analysis was performed to detect NFATc1 abundance in the cytoplasmic and nuclear fractions, which was expressed as the ratio of NFATc1 level in control cells treated with RANKL alone. PCNA and β‐tubulin were used as internal reference proteins for the cytoplasmic and nuclear fractions, respectively.

### 2.8 Immunofluorescence analysis

To determine p65 nuclear translocation, BMMs were treated with 100 μmol/L STA for 4 hours, followed by stimulation with 50 ng/mL RANKL for 15 minutes. For determining NFATc1 nuclear translocation, BMMs were stimulated with 25 ng/mL M‐CSF and 50 ng/mL RANKL in the presence or absence of 100 μmol/L STA for 0, 2 or 4 days. The cells were fixed with 4% PFA for 15 minutes, permeabilized with 0.3% Triton X‐100 for 20 minutes and non‐specifically blocked in 1% BSA for 30 minutes. The fixed cells were washed and incubated overnight with the primary antibody against NFATc1 or p65. Next, the cells were incubated with an appropriate fluorescence‐conjugated secondary antibody (Invitrogen) for 120 minutes, stained with rhodamine‐conjugated phalloidin for 20 minutes and mounted in Fluoroshield with DAPI (Sigma‐Aldrich). Fluorescent images were captured using a fluorescence microscope (Leica).

### 2.9 Luciferase assay

RAW264.7 cells were transfected with NF‐κB luciferase reporter (Beyotime, #D2206) and Renilla luciferase reporter (Beyotime, #D2762) for 8 hours by Lipofectamine2000 (Invitrogen) according to the manufacturer's instructions. Next, cells were treated with 50ng/mL RANKL in the presence or absence of 100 μmol/L STA for 24 or 48 hours. Then, the cells were lysed and the luciferase activities were detected by a Dual‐Luciferase Reporter Assay System (Promega).

### 2.10 Mouse model of LPS‐induced tibial osteolysis

All animal care and experimental protocols were designed and performed in compliance with National Institutes of Health (NIH) guide for the care and use of Laboratory Animals and the Guide of the Animal Care Committee of Zhejiang University. Thirty 8‐week‐old male C57BL/6 mice weighing 18‐22 g were purchased from Experimental Animal Center of Zhejiang University. A mouse model of LPS‐induced tibial osteolysis was established, as described previously,^24^, ^25^ to determine the effects of STA on inflammatory bone loss in vivo. The mice were acclimatized to laboratory conditions for 1 week and were randomly divided into the following three experimental groups (n = 5 per group): sham, LPS and LPS + STA groups. The mice in the LPS and LPS + STA groups were intraperitoneally injected with 5 mg/kg body weight LPS (Sigma‐Aldrich) on days 1 and 4, whereas the mice in the sham group were injected with PBS as a control. Next, the mice in the LPS + STA group were intragastrically administered 10 mg/kg STA every day for 7 days. The mice in the sham and LPS groups were intragastrically administered PBS as a control. STA doses were determined according to previous studies.^17^, ^26^ On day 7, all the mice were killed and their tibias were harvested for subsequent analysis.

### 2.11 Micro‐CT scanning

Fixed tibias were collected and analysed (n = 5 per group) using a high‐resolution micro‐CT scanner (SkyScan 1072; SkyScan) at an isometric resolution of 9 μm. The scanning energy of X‐ray was set at 70 kV and 80 μA. Three‐dimensional (3D) reconstruction was performed with micro‐CT data. Structure model index (SMI), bone surface/volume ratio (BS/BV), bone volume/tissue volume (BV/TV), trabecular thickness (Tb.Th), trabecular number (Tb.N) and trabecular separation (Tb.Sp) were quantified and analysed as described previously.^27^

### 2.12 Histological and immunohistochemical analyses

The mouse tibias (n = 5 per group) were fixed in 4% PFA for 2 days, decalcified in 10% EDTA (pH = 7.4) for 4 weeks and embedded in paraffin. Next, the tibias were cut into 4‐μm‐thick histological sections for performing haematoxylin and eosin (H&E) and TRAP staining. The sections were photographed and analysed using a light microscope (TE2000‐S; Nikon). BV/TV, Tb.Th, Tb.N, number of osteoclasts per bone surface (N.Oc/BS) and surface area of osteoclasts per bone surface (OcS/BS) were assessed for each section. Active p65 and NFATc1 levels surrounding the trabecular bone were determined by performing immunohistochemical staining of the histological sections. Rehydrated histological sections were blocked by incubating with 10% goat serum for 30 minutes, followed by overnight incubation at 4°C with the primary antibodies against active p65 (Millipore) and NFATc1 (Abcam) at 4°C overnight. After washing, the sections were incubated with a goat antimouse secondary antibody (Beyotime) for 1 hours at 37°C. Colour development was achieved using SignalStain DAB Substrate Kit (Cell Signaling Technology), according to the manufacturer's protocol. The number of p65‐ or NFATc1‐positive cells per bone surface was measured for each section by using a microscope.

### 2.13 Statistical analysis

Data were expressed as mean ± SEM of at least three independent experiments. Statistical analysis was performed using Prism 6.01 (GraphPad Software). Differences between two groups were compared using a two‐tailed unpaired Student's *t* test. One‐way ANOVA with post hoc Newman‐Keuls test was performed to analyse differences in multiple group comparisons. *P* < 0.05 was considered to be statistically significant.

## 3 RESULTS

### 3.1 STA inhibits RANKL‐induced osteoclast formation in vitro

**Figure 1:**
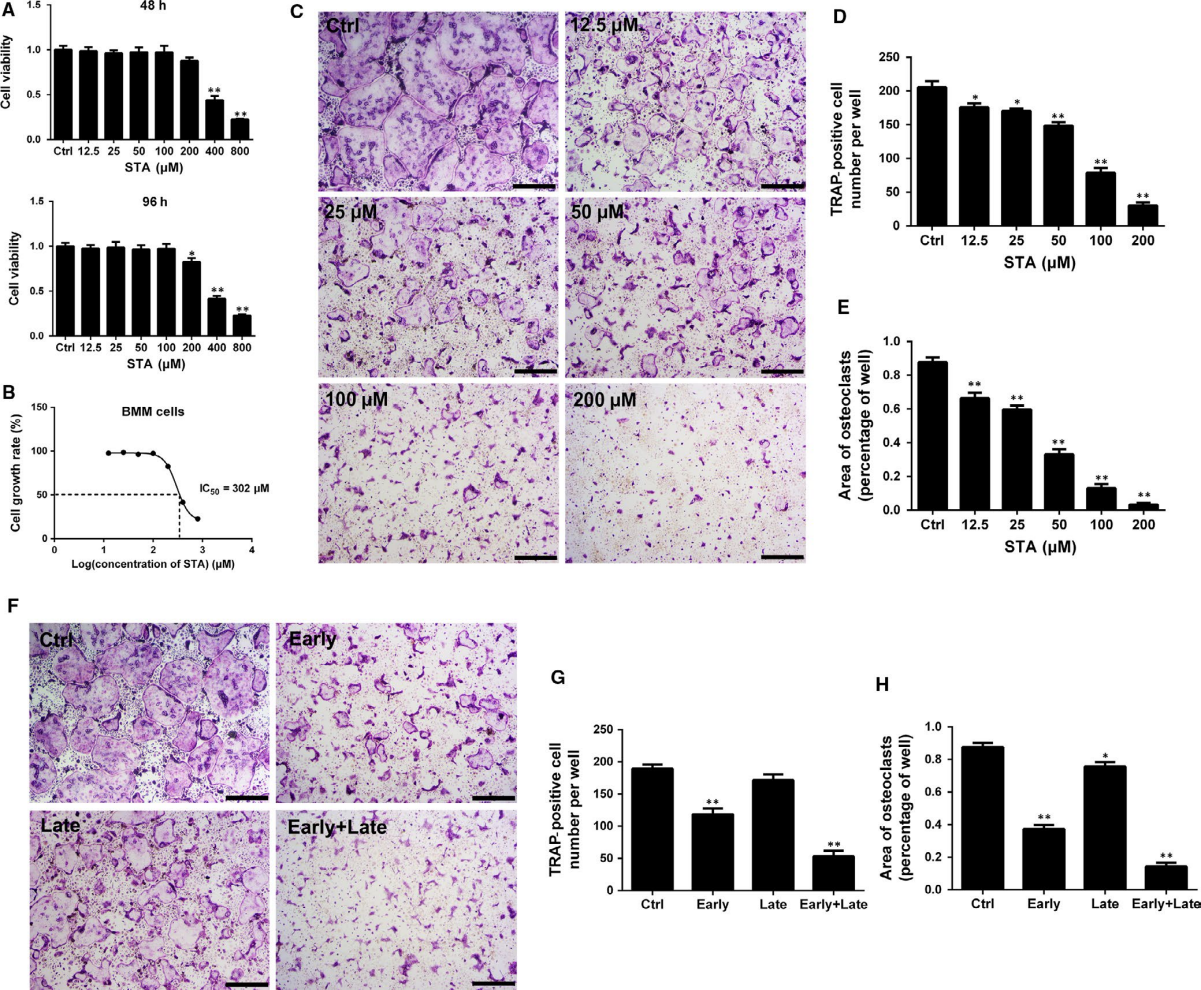
STA inhibits RANKL‐induced osteoclast formation in vitro. A, Viability of BMMs exposed to the indicated STA concentrations was measured by performing the CCK‐8 assay at 48 and 96 h. B, The IC_50_ values of STA against BMMs. C, BMMs were treated with 25 ng/mL M‐CSF, 50 ng/mL RANKL and the indicated STA concentrations for 5 d. The cells were stained for TRAP and were photographed; scale bar = 500 μm. The (D) number and (E) area of TRAP‐positive cells were analysed. (F) BMMs were stimulated with 25 ng/mL M‐CSF and 50 ng/mL RANKL for 5 d, and 100 μmol/L STA was added at different stages during the 5‐day period. The cells were stained for TRAP and were photographed; scale bar = 500 μm. The (G) number and (H) area of TRAP‐positive cells were analysed. ^*^ *P* < 0.05 and ^**^ *P* < 0.01 compared with the controls

The potential cytotoxic effect of STA on BMMs was determined by performing the CCK‐8 assay. Treatment with 100 μmol/L STA did not affect BMM viability (Figure 1A). The half maximal inhibitory concentration (IC_50_) of STA was 302 μmol/L at 96 hours (Figure 1B).

To examine the effect of STA on osteoclast formation, BMMs were treated with M‐CSF, RANKL and different STA concentrations (0, 12.5, 25, 50, 100 and 200 μmol/L) for 5 days. After culturing for 5 days, a large number of TRAP‐positive multinucleated osteoclasts were observed in the control group. However, STA decreased the number and area of TRAP‐positive multinucleated osteoclasts (Figure 1C‐E), indicating that STA inhibited osteoclast formation in a dose‐dependent manner.

To clarify at which stage of osteoclastogenesis STA exerted its inhibitory effect, BMMs were treated with 100 μmol/L STA on days 1‐3 (early stage), 3‐5 (late stage) and 1‐5 (early + late stage). Treatment of BMMs with 100 µmol/L STA in the early stage markedly inhibited osteoclast formation (Figure 1F). However, STA treatment in the late stage did not effectively suppress osteoclast formation. A small but significant decrease in osteoclast area was observed for BMMs treated with STA in the late stage; however, no difference was observed in osteoclast number. Together, these results indicate that prolonged exposure to STA dose‐dependently suppresses osteoclast formation, especially in the early stage and the entire period of RANKL stimulation.

### 3.2 STA attenuates osteoclastic bone resorption and F‐actin ring formation in vitro

**Figure 2:**
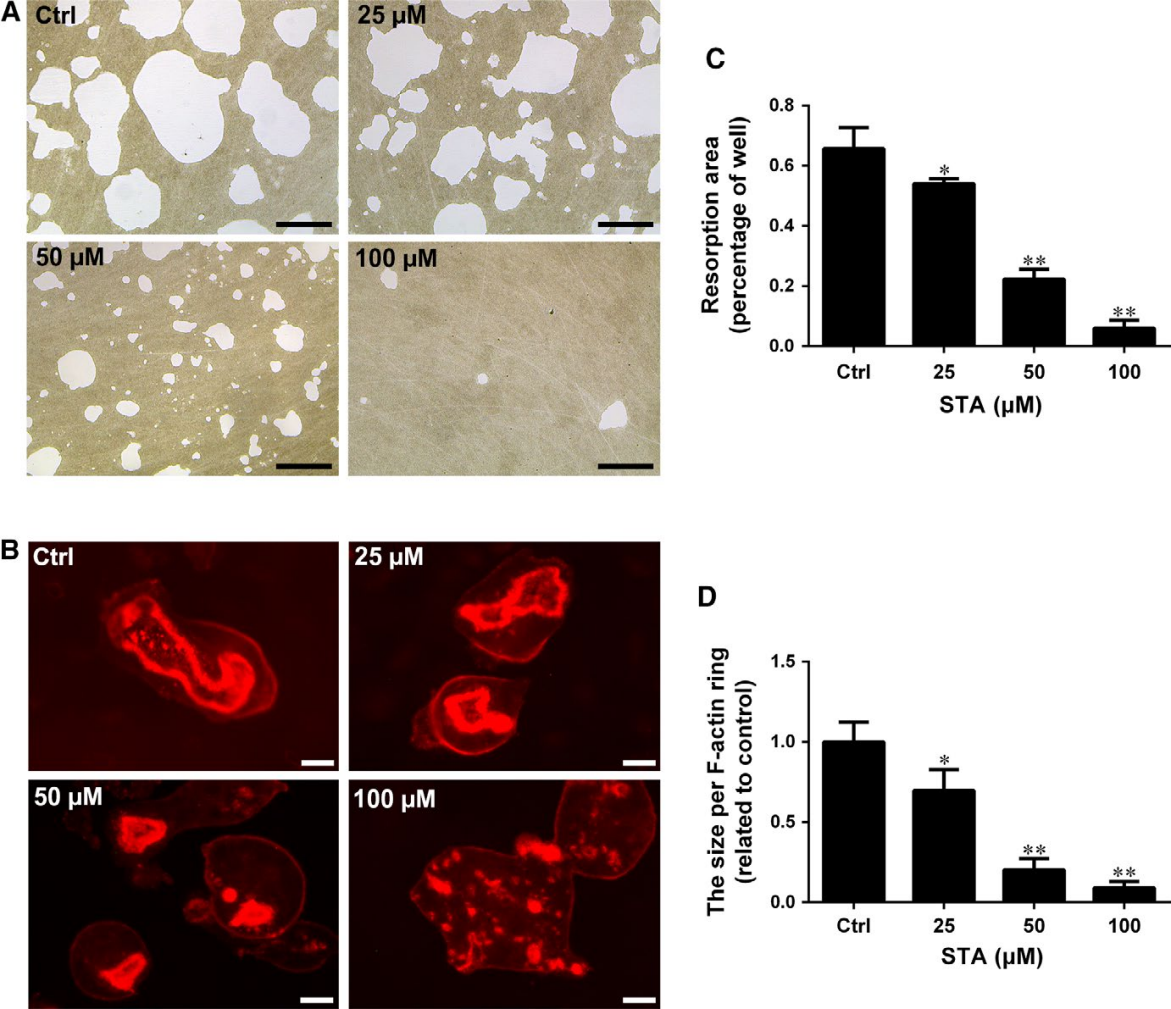
STA attenuates osteoclastic bone resorption and F‐actin ring formation in vitro. An equal number of BMM‐derived mature osteoclasts were seeded into the Osteo Assay Plate and were treated with the indicated STA concentrations for 2 d. A, The cells were completely removed by incubation with NaClO. Representative light microscope images of resorption pits are shown; scale bar = 100 μm. B, Resorption pit area was quantified using the ImageJ software. C, The cells were fixed and stained with rhodamine‐conjugated phalloidin for observing F‐actin rings. Representative fluorescence microscope images are shown; scale bar = 20 μm. D, The size per F‐actin ring was measured using the ImageJ software. ^*^ *P* < 0.05 and ^**^ *P* < 0.01 compared with the controls

Given that STA markedly suppressed osteoclast formation, we assessed its effect on the resorptive function of mature osteoclasts. Mature osteoclasts were plated onto the Osteo Assay Plate and were treated with the indicated STA doses in an osteoclastogenic‐inducing medium for 2 days. Optical images showed extensive resorption of the bone surface by osteoclasts in the control group (Figure 2A). In contrast, bone resorption area was significantly reduced to 54% and 23% after treatment with 25 and 50 μmol/L STA, respectively; moreover, rare resorption pit was observed in the plate containing cells treated with 100 μmol/L STA (Figure 2A,B).

A tight F‐actin ring is a prerequisite for osteoclastic bone resorption and is an observable marker of mature osteoclasts.^28^, ^29^ Immunofluorescence analysis indicated that STA disrupted the morphology and size of F‐actin rings (Figure 2C). Statistically, STA decreased the size of F‐actin rings in a dose‐dependent manner (Figure 2D). Together, our results suggest that STA suppresses the bone resorptive activity and F‐actin ring formation of mature osteoclasts in vitro.

### 3.3 STA suppresses RANKL‐induced osteoclast‐related gene expression

**Figure 3:**
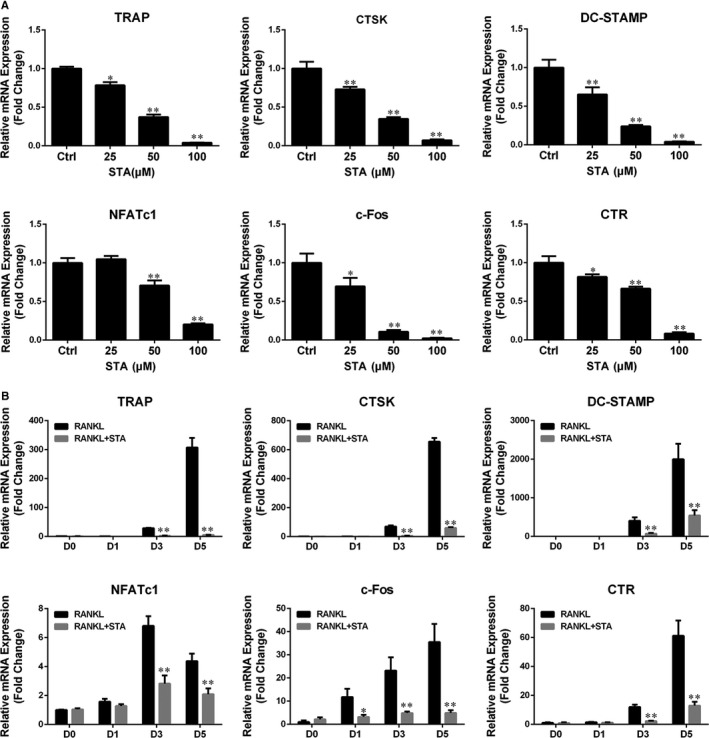
STA suppresses RANKL‐induced expression of osteoclast‐specific genes. A, BMMs were cultured with 25 ng/mL M‐CSF, 50 ng/mL RANKL and the indicated STA concentrations for 5 d. B, BMMs were cultured with 25 ng/mL M‐CSF and 50 ng/mL RANKL, with or without 100 μmol/L STA, for 0, 1, 3 or 5 days. The mRNA expression of the osteoclast‐specific genes TRAP, CTSK, DC‐STAMP, NFATc1, c‐Fos and CTR was determined by performing qPCR. ^*^ *P* < 0.05 and ^**^ *P* < 0.01 compared with the controls

With the stimulation of RANKL, several specific genes are up‐regulated during osteoclast differentiation. Therefore, qPCR was performed to investigate the inhibitory effect of STA on the expression of osteoclast differentiation‐related genes. Our results showed that RANKL dramatically up‐regulated the expression levels of all the evaluated genes, including TRAP, CTSK, CTR, DC‐STAMP, c‐Fos and NFATc1, whereas STA substantially suppressed the expression of these genes in a dose‐ and time‐dependent manner (Figure 3A,B). These results confirm that STA inhibits osteoclast formation and osteoclast‐specific gene expression in vitro.

### 3.4 STA inhibits RANKL‐induced NF‐κB and Akt signalling

**Figure 4:**
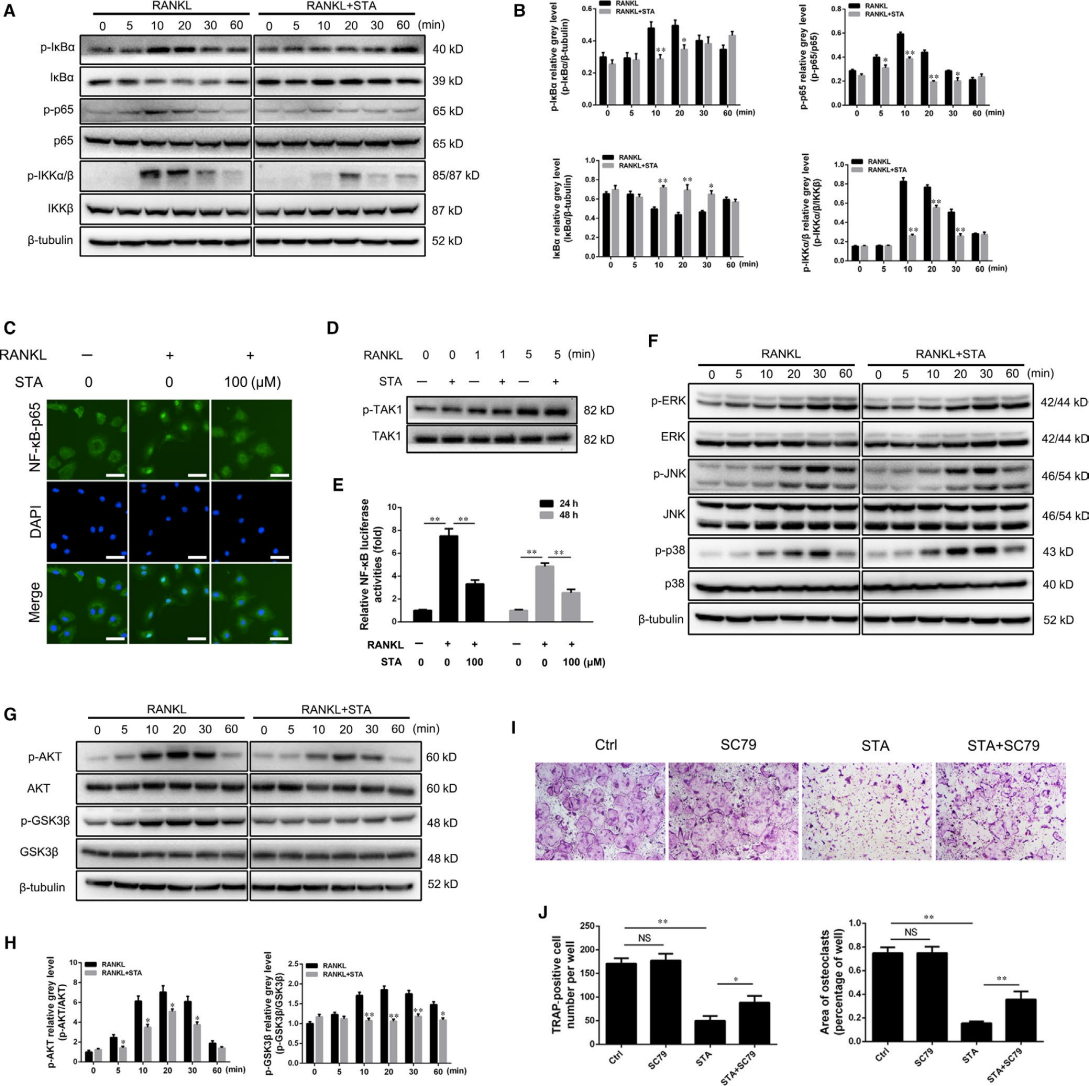
STA inhibits osteoclastogenesis by suppressing RANKL‐induced activation of NF‐κB and Akt signalling. (A, D, F and G) BMMs were pretreated with 100 μmol/L STA or DMSO for 4 h, followed by stimulation with 50 ng/mL RANKL for the indicated period. Whole‐cell lysates were analysed by performing Western blotting analysis. (B and H) The grey levels of phosphorylated p65, IKKα/β, Akt and GSK3β were quantified and normalized relative to their total protein counterparts. The grey levels of p‐IκBα and IκBα were normalized to β‐tubulin. C, BMMs were pretreated with 100 μmol/L STA or DMSO for 4 h, followed by stimulation with 50 ng/mL RANKL for 15 min. NF‐κB p65 localization was visualized by performing immunofluorescence staining. Nuclei were counterstained with DAPI. NF‐κB p65 (green) and nuclei (blue) were examined under a fluorescence microscope; scale bar = 20 μm. E, Relative NF‐κB luciferase activities in RAW264.7 cells treated with RANKL in the presence or absence of 100 μmol/L STA for 24 h or 48 h. I, BMMs were cultured with 25 ng/mL M‐CSF and 50 ng/mL RANKL, with or without STA for 5 d, in the presence or absence of SC79. Cells were fixed and TRAP staining was performed. J, The number and area of TRAP‐positive cells were quantified. ^*^ *P* < 0.05 and ^**^ *P* < 0.01 compared with the cells treated with RANKL alone

To determine the exact mechanisms through which STA inhibited osteoclastogenesis, we investigated the effects of STA on NF‐κB, MAPK and Akt pathways in RANKL signalling. The NF‐κB pathway is an important signalling pathway involved in the early stage of RANKL‐induced osteoclastogenesis.^30^ Treatment with 100 μmol/L STA effectively suppressed RANKL‐induced IκBα phosphorylation and degradation (Figure 4A). Moreover, results of Western blotting and immunofluorescence analyses showed that STA attenuated NF‐κB p65 phosphorylation and nuclear translocation (Figure 4A‐C). To determine the molecular target of STA, we explored upstream molecules involved in the RANKL‐induced NF‐κB pathway. We observed that STA suppressed IKKα/β phosphorylation (Figure 4A,B) but did not affect TAK1 phosphorylation (Figure 4D and Figure S1). Next, we compared the efficiency of STA to SC‐514, a well‐established NF‐κB inhibitor. Our data showed when compared to SC‐514, STA exerted a similar inhibitory effect on RANKL‐induced NF‐κB signalling, and even slightly better inhibition on osteoclast differentiation (Figures S2 and S3). To further determine the effect of STA on NF‐κB activity in longer time‐points, we performed NF‐κB luciferase gene reporter assay. Treatment with 100 μmol/L STA was shown to inhibit NF‐κB activity at 24 and 48 hours (Figure 4E). The MAPK pathways (including ERK, JNK and p38 pathways) also play a crucial role in RANKL‐induced osteoclastogenesis.^31^ We observed that STA did not affect RANKL‐induced activation of the ERK, JNK and p38 pathways (Figure 4F and Figure S4), indicating that it did not affect the MAPK pathways.

As the Akt‐GSK3β signalling pathway is important for RANKL‐induced osteoclast differentiation,^32^ we next investigated whether STA affected the activation of Akt and GSK3β. As shown in Figure 4G, STA inhibited RANKL‐induced phosphorylation of Akt and GSK3β. Quantitative analysis confirmed these observations (Figure 4H). To further confirm these results, we determined whether combining SC79 (an AKT agonist) can rescue osteoclast differentiation. As expected, osteoclast differentiation was suppressed in cells treated with STA alone. However, in cells co‐treated with SC79, the impaired osteoclastogenesis was partly rescued, as indicated by an increased number and larger size of osteoclasts (Figure 4I,J).

**Figure 5:**
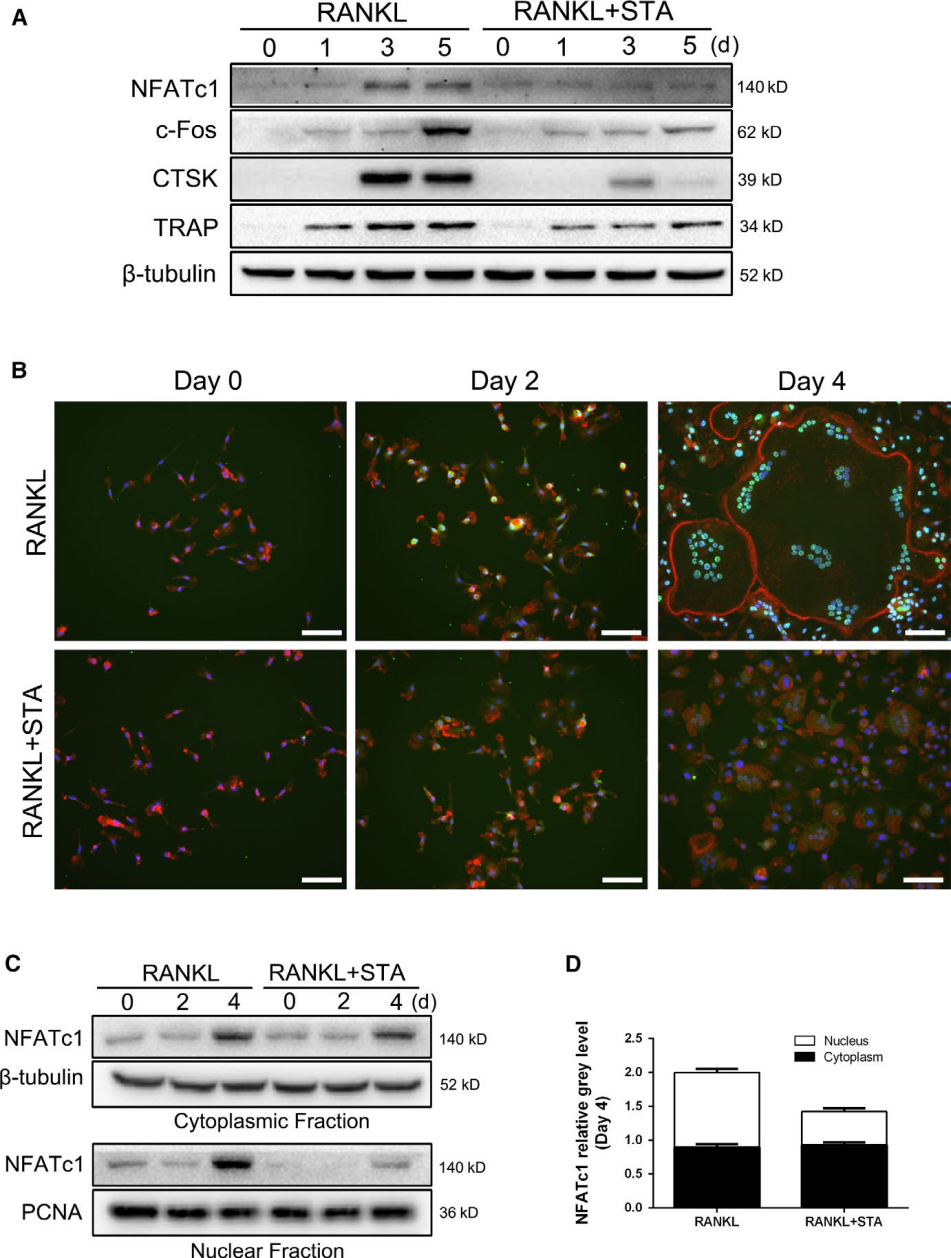
STA attenuates RANKL‐induced NFATc1 activation and nuclear translocation. A, BMMs were cultured with 25 ng/mL M‐CSF and 50 ng/mL RANKL, with or without 100 μmol/L STA, for 0, 1, 3 or 5 d. Whole‐cell lysates were analysed by performing Western blotting analysis. B, BMMs were cultured on glass coverslips and were treated with 25 ng/mL M‐CSF and 50 ng/mL RANKL, with or without 100 μmol/L STA, for 0, 2 and 4 d. NFATc1 localization was visualized by performing immunofluorescence staining. Nuclei and F‐actin rings were counterstained with DAPI and rhodamine‐conjugated phalloidin, respectively. NFATc1 (green), nuclei (blue) and F‐actin rings (red) were detected using a fluorescence microscope; scale bar = 100 μm. C, The cytoplasmic and nuclear fractions of the BMMs treated with 25 ng/mL M‐CSF, 50 ng/mL RANKL and 100 μmol/L STA for 0, 2 and 4 d were analysed by performing Western blotting analysis. PCNA and β‐tubulin were used as nuclear and cytoplasmic loading controls, respectively. D, The relative abundance of NFATc1 in the nuclear and cytoplasmic fractions was normalized to loading controls and was expressed as the ratio of the cytoplasmic NFATc1 in the cells treated with RANKL alone

RANKL‐induced activation of the NF‐κB and Akt pathways leads to the induction and activation of NFATc1, which is the master transcription factor involved in RANKL‐induced osteoclastogenesis.^33^, ^34^ Therefore, we explored whether STA altered NFATc1 expression after RANKL stimulation. STA suppressed NFATc1 protein expression levels in the whole‐cell lysates of BMMs stimulated with RANKL for 3 and 5 days (Figure 5A). Similarly, STA reduced the protein expression levels of c‐Fos, TRAP and CTSK, all of which are the downstream transcription targets of NFATc1 and crucial for osteoclast formation and bone resorption (Figure 5A). NFATc1 nuclear translocation plays an important role in regulating the transcription of osteoclast‐related genes.^35^ Immunofluorescence and immunoblotting analyses showed that RANKL induced NFATc1 nuclear translocation during osteoclast formation in a time‐dependent manner, whereas STA markedly attenuated NFATc1 nuclear translocation (Figure 5B‐D). Together, these findings suggest that STA impairs osteoclastogenesis by suppressing the activation of NF‐κB signalling and by preventing the induction and nuclear translocation of NFATc1.

### 3.5 STA prevents LPS‐induced bone loss in a mouse model

**Figure 6:**
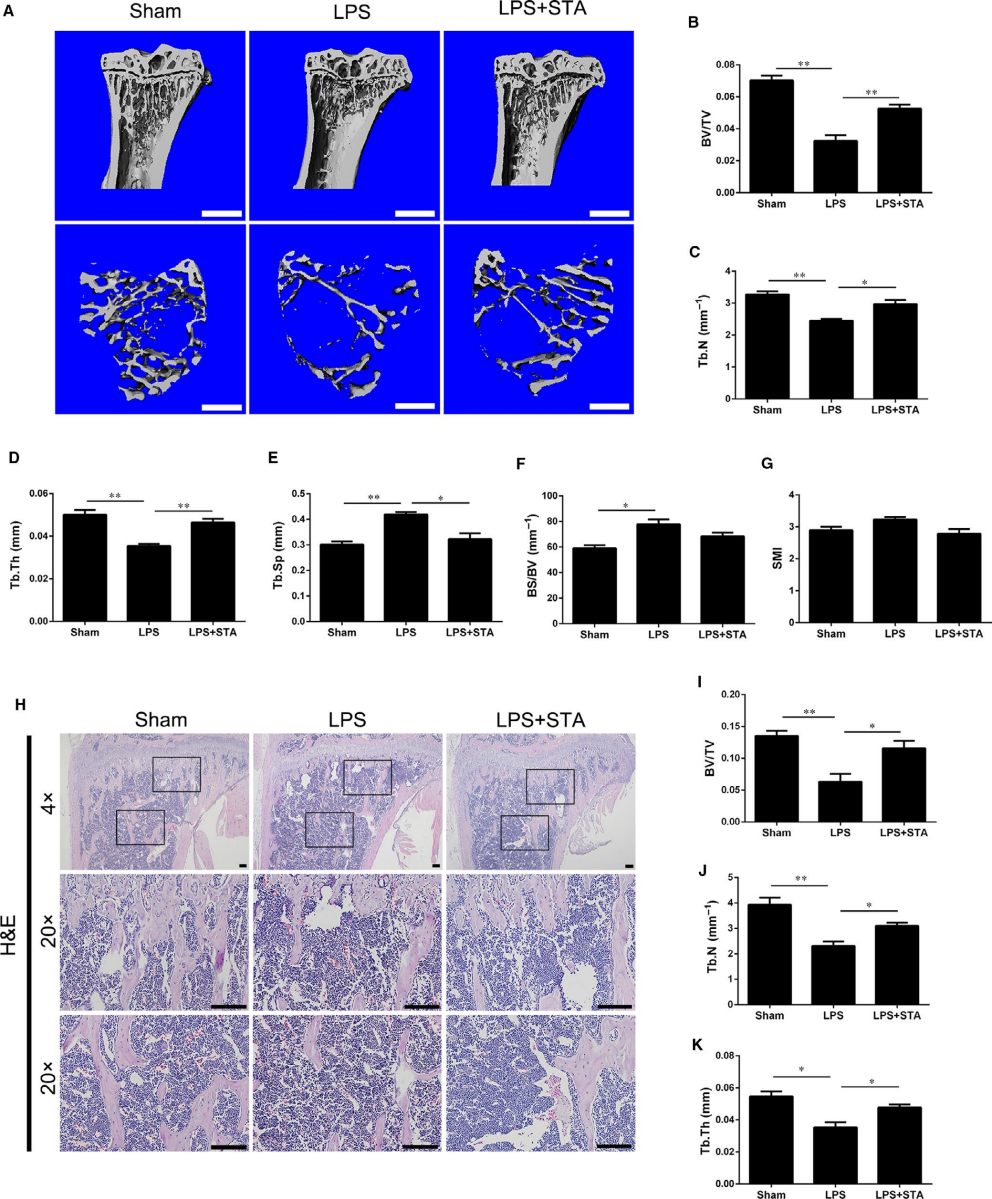
STA protects against LPS‐induced bone loss in vivo. A, Representative micro‐CT 3D reconstructed images of the tibias of the mice in the sham, LPS and LPS + STA groups; scale bar = 1 mm. B‐G, Quantitative analysis of the BV/TV, Tb.Sp, Tb.N, Tb.Th, BS/BV and SMI was performed using the micro‐CT data. H, Representative images of H&E staining of the tibial histological sections of the mice in the sham, LPS and LPS + STA groups; scale bar = 100 μm. I‐K, Quantitative analysis of the BV/TV, Tb.N and Tb.Th was performed. ^*^ *P* < 0.05 and ^**^ *P* < 0.01 compared with the mice in the LPS group

To investigate the potential protective effects of STA against pathological osteolysis, we established a murine model of LPS‐induced inflammatory bone loss. The mice were intraperitoneally injected with LPS on days 1 and 4, with or without the intragastric administration of STA every day. After 7 days, the mouse tibias were collected and analysed by performing the micro‐CT and histological analyses. Micro‐CT data indicated that the mice in the LPS group suffered from extensive bone loss in the tibias compared with the mice in the sham group (Figure 6A). Quantitative analysis of the bone parameters indicated that BV/TV, Tb.Th and Tb.N were significantly lower and Tb.Sp and BS/BV were higher in the mice in the LPS group than those in the sham group (Figure 6B‐G). In contrast, the mice in the LPS + STA group showed a marked increase in BV/TV, Tb.Th and Tb.N and a pronounced decrease in Tb.Sp compared with the mice in the LPS group (Figure 6B‐E), indicating that STA exerted a protective effect against LPS‐induced bone loss. Moreover, the mice in the LPS + STA group showed a slight decrease in BS/BV and SMI compared with the mice in the LPS group, although the difference was not statistically significant (Figure 6F,G). Histological analysis also confirmed the protective effect of STA against LPS‐induced bone loss. H&E staining showed a significant bone loss in the mice in the LPS group compared with those in the sham group. However, the mice in the LPS + STA group showed restoration of the BV/TV, Tb.Th and Tb.N values (Figure 6H‐K), indicating that STA effectively attenuated LPS‐induced bone loss. Thus, these data indicate that STA protects against LPS‐induced bone loss in vivo.

### 3.6 STA alleviates bone loss by inhibiting osteoclast activity through NF‐κB–NFATc1 signalling

**Figure 7:**
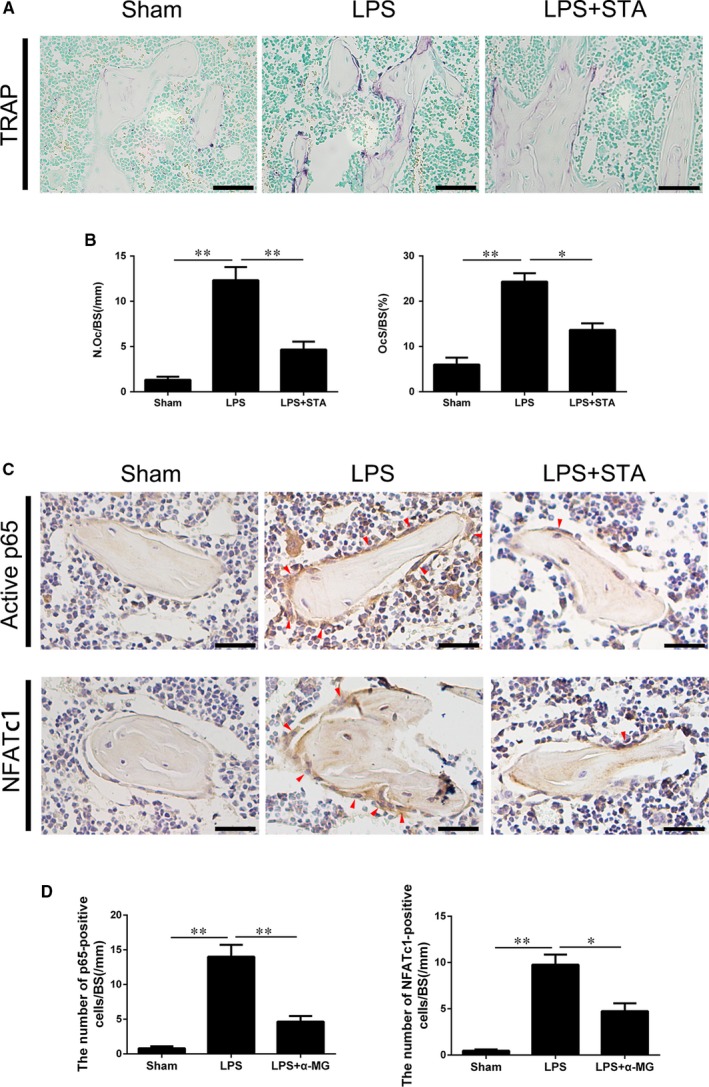
STA attenuates bone loss by inhibiting osteoclast activity through NF‐κB–NFATc1 signalling. A, Representative images of TRAP staining of the tibial histological sections of the mice in the sham, LPS and LPS + STA groups; scale bar = 50 μm. B, Quantitative analysis of the N.Oc/BS and OcS/BS (%) was performed. C, Immunohistochemical staining of active p65 and NFATc1 (red arrowheads) in the tibias of the mice in the sham, LPS and LPS + STA groups. D, Active p65‐ or NFATc1‐positive cells per bone surface surrounding the trabecular bone were quantified; scale bar = 30 μm. ^*^ *P* < 0.05 and ^**^ *P* < 0.01 compared with the mice in the LPS group

Considering the importance of osteoclasts in inflammatory bone loss, we performed TRAP staining to investigate the effect of STA on osteoclast formation and activity in vivo. LPS induced the formation of TRAP‐positive osteoclasts along the trabecular bone surface, whereas STA decreased the number of osteoclasts (Figure 7A). Histomorphometric analysis by determining OcS/BS and N.Oc/BS indicated that STA attenuated LPS‐induced excessive osteoclast formation (Figure 7B).

As STA inhibited NF‐κB–NFATc1 signalling in vitro, we further evaluate its effect in vivo. Immunohistochemical staining showed that LPS elevated activated p65 and NFATc1 levels in osteoclasts surrounding the trabecular bone and that STA significantly inhibited the increase in NF‐κB and NFATc1 activities (Figure 7C,D). Quantitative analysis of the positive cell number per bone surface also confirmed these observations (Figure 7C,D). Together, these results suggest that STA protects against LPS‐induced bone loss by inhibiting osteoclast activity through NF‐κB–NFATc1 signalling.

## 4 DISCUSSION

An imbalance in bone homeostasis caused by an increase in the number and/or activity of osteoclasts leads to pathological bone destruction, which is a characteristic of many osteolytic diseases, including rheumatoid arthritis, periprosthetic osteolysis, periodontitis and osteoporosis.^36^, ^37^ Therefore, osteoclasts are the key targets for developing potential new drugs for treating these diseases. In the previous two decades, significant advances have been made in osteolytic disease treatment by using osteoclast‐targeting therapies such as bisphosphonates, oestrogens and denosumab. However, these therapies are associated with high cost and side effects, including gastrointestinal toxicity, jaw osteonecrosis, atypical fractures, breast cancer and thromboembolism, which limit their clinical application.^38^, ^39^ The present study is the first to show that STA inhibits osteoclast formation and bone resorption by suppressing RANKL‐induced activation of NF‐κB–NFATc1 signalling in vitro. In addition, this study showed that STA attenuates LPS‐induced bone loss in a murine model, suggesting its potential for treating inflammatory bone loss and osteoclast‐related diseases.

Activation of RANK–RANKL signalling plays an important role in inflammatory bone erosion that is observed in rheumatoid arthritis, periodontitis and aseptic loosening of orthopaedic implants.^40^, ^41^, ^42^ The binding of RANKL to its receptor RANK on osteoclast precursor cells activates downstream signalling pathways, including the NF‐κB and MAPK pathways, which are important in osteoclast differentiation and function. Interestingly, we observed that STA inhibited RANKL‐induced phosphorylation and activation of the NF‐κB pathway without affecting the MAPK pathways, including the ERK, JNK and p38 pathways. To elucidate the molecular mechanism through which STA inhibited the NF‐κB pathway, we investigated signalling proteins associated with this pathway. After RANKL stimulation, the RANK receptor associates its cytoplasmic domain with TRAF6 and forms a signalling complex containing RANK, TAK1 and TAK1‐binding protein‐2, resulting in TAK1 activation. The activated TAK1 then phosphorylates the IKK complex to initiate the NF‐κB pathway.^43^ However, inactive NF‐κB subunits are retained in the cytoplasm by inhibitory IκB. The activated IKK complex catalyses the phosphorylation and subsequent degradation of IκB, which releases NF‐κB p65/RelA that is translocated to the nucleus to initiate target gene transcription.^30^, ^43^ In the present study, we found that STA inhibited IKKα/β activation without affecting TAK1 phosphorylation and attenuated IκBα phosphorylation and degradation, thereby suppressing NF‐κB p65 activation and nuclear translocation. These findings suggest that STA impairs RANKL‐induced osteoclastogenesis by inhibiting IKK activation.

NFATc1 is the key target gene of NF‐κB in the early stage of osteoclastogenesis. After activation, NF‐κB translocates into the nucleus and binds to the NFATc1 promoter, switching on the initial induction of NFATc1.^7^, ^8^ The importance of NFATc1 in osteoclastogenesis is suggested by an in vitro observation that NFATc1^−/−^ embryonic stem cells do not differentiate into osteoclasts after RANKL stimulation.^44^ NFATc1 is the master transcription factor that regulates the expression of osteoclast‐specific genes, including TRAP, CTSK, DC‐STAMP and CTR.^33^, ^45^ Our findings suggested that the inhibition of the NF‐κB pathway impaired the induction and nuclear translocation of NFATc1 during osteoclast differentiation. In addition, STA suppressed the mRNA and protein expression of osteoclast‐specific genes in a dose‐dependent manner. These results suggest that the inhibitory effect of STA on osteoclastogenesis could be partly attributed to inhibition of early NF‐κB signalling and subsequent down‐regulation of NFATc1 expression.

Previous studies have established that Akt‐GSK3β‐NFATc1 signalling cascade is also critical for osteoclast formation.^32^, ^34^, ^46^ The stimulation of RANKL and M‐CSF activates Akt, which in turn phosphorylates GSK3β and inhibits its kinase activity. This increased level of phospho‐GSK3β (inactive form of GSK3β) can enhance the phosphorylation of NFATc1 and promote the nuclear localization of NFATc1, resulting in osteoclastogenesis. In this study, STA suppressed the phosphorylation of Akt and GSK3β during osteoclast differentiation, and an Akt agonist (SC79) could partly reverse the inhibited osteoclast differentiation, suggesting the inhibitory effect of STA on osteoclast differentiation is partly due to its inhibition on Akt‐GSK3β‐NFATc1 signalling pathway.

Consistent with its anti‐osteoclastogenic effect in vitro, STA prevented LPS‐induced inflammatory bone loss in vivo. LPS, a cell wall component of gram‐negative bacteria, induces inflammatory bone loss by recruiting inflammatory cells, promoting proinflammatory cytokine secretion, increasing osteoclast number and activating osteoclastic bone resorption. Micro‐CT analysis performed in the present study showed that STA treatment for the LPS‐injected mice increased BV/TV, Tb.Th and Tb.N and decreased Tb.Sp, indicating that STA partly restored LPS‐induced severe bone loss. Bone histomorphometry analysis showed that STA markedly reduced LPS‐induced TRAP‐positive osteoclast formation and bone erosion in vivo. Furthermore, STA markedly inhibited NF‐κB and NFATc1 activities in vivo, which was consistent with the in vitro results. These results obtained here provided evidence that STA exerts a therapeutic effect on inflammatory bone loss and this effect of STA is mainly mediated by suppressing osteoclast activity and NF‐κB–NFATc1 signalling.

Despite these promising results, our study has some limitations. First, LPS‐induced inflammatory bone loss in vivo is a complex process involving many cell types. In the present study, we investigate the effects of STA on osteoclast formation and osteoclastic bone resorption. However, we cannot exclude the possibility that STA might affect osteoblastic bone formation. Therefore, further studies are needed to assess the effect of STA on osteoblastic bone formation. Second, our study only focused on LPS‐induced pathological bone loss, which is dominated by excessive osteoclast activity. Therefore, additional studies assessing the effects of STA on the normal bone or other pathological conditions are needed to completely elucidate the effects and mechanisms of action of STA in bone remodelling.

In summary, the present study is the first to show that STA inhibits both osteoclastogenesis and bone resorption in vitro. These inhibitory effects of STA are mediated by the inhibition of the RANKL‐induced NF‐κB and Akt signalling pathways, leading to suppression of the induction, accumulation and nuclear translocation of NFATc1. Furthermore, STA exerts protective effects against LPS‐induced inflammatory bone loss in vivo, suggesting its potential for preventing or treating osteoclast‐related diseases.

## CONFLICT OF INTEREST

The authors confirm that there are no conflicts of interest.

## AUTHOR CONTRIBUTION

Jiahong Meng, Chenhe Zhou and Wenkan Zhang contributed equally to this study. Jiahong Meng, Shigui Yan and Weiqi Yan designed this research; Jiahong Meng, Chenhe Zhou, Guangyao Jiang and Jianqiao Hong performed in vitro research; Jiahong Meng, Bin Hu and Bin He performed the animal study; Wei Wang, Sihao Li and Jiamin He analysed the data; Jiahong Meng and Yangxin Wang wrote the paper; experiments were performed under the supervision of Shigui Yan and Weiqi Yan.

## Data Availability

All data used to support the findings of this study are available from the corresponding authors upon request.
