# Supplementary figures and images for: Stachydrine prevents LPS‐induced bone loss by inhibiting osteoclastogenesis via NF‐κB and Akt signalling
Source: J Cell Mol Med. 2019 Jul 21;23(10):6730–43. doi: 10.1111/jcmm.14551 (PMC6787569; doi:10.1111/jcmm.14551)

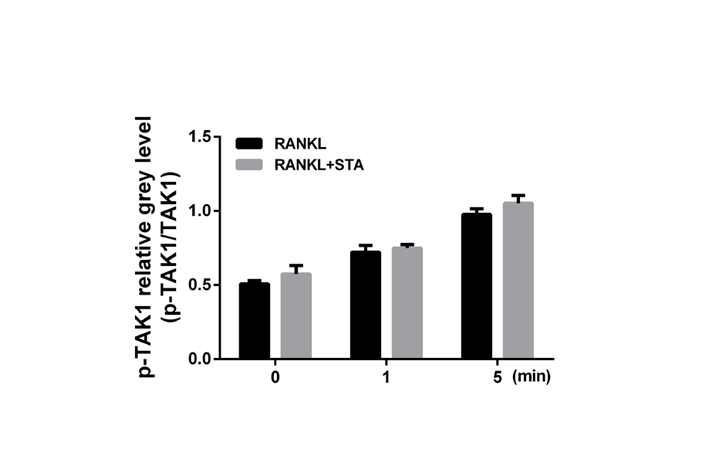

Supplement: Supplementary file 1 [file JCMM-23-6730-s001.tif]

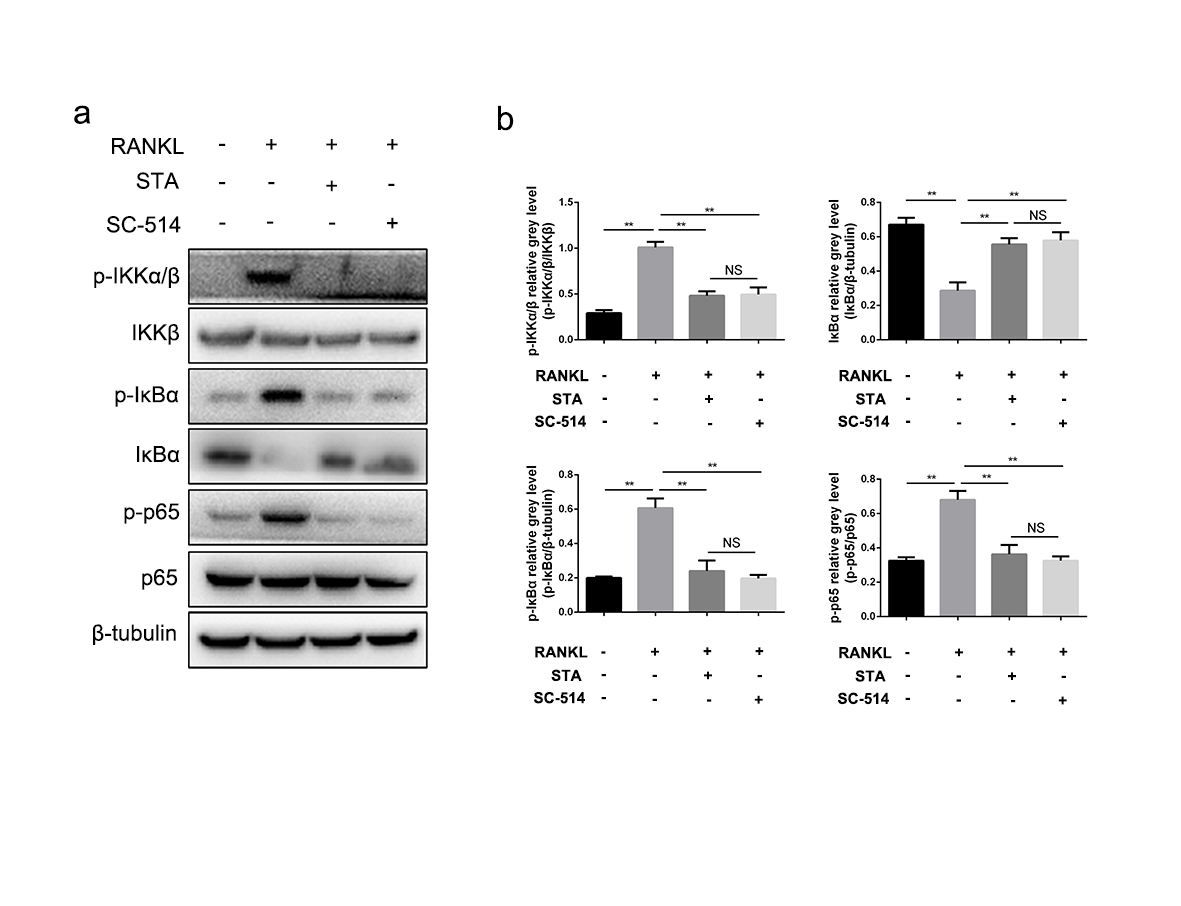

Supplement: Supplementary file 2 [file JCMM-23-6730-s002.tif]

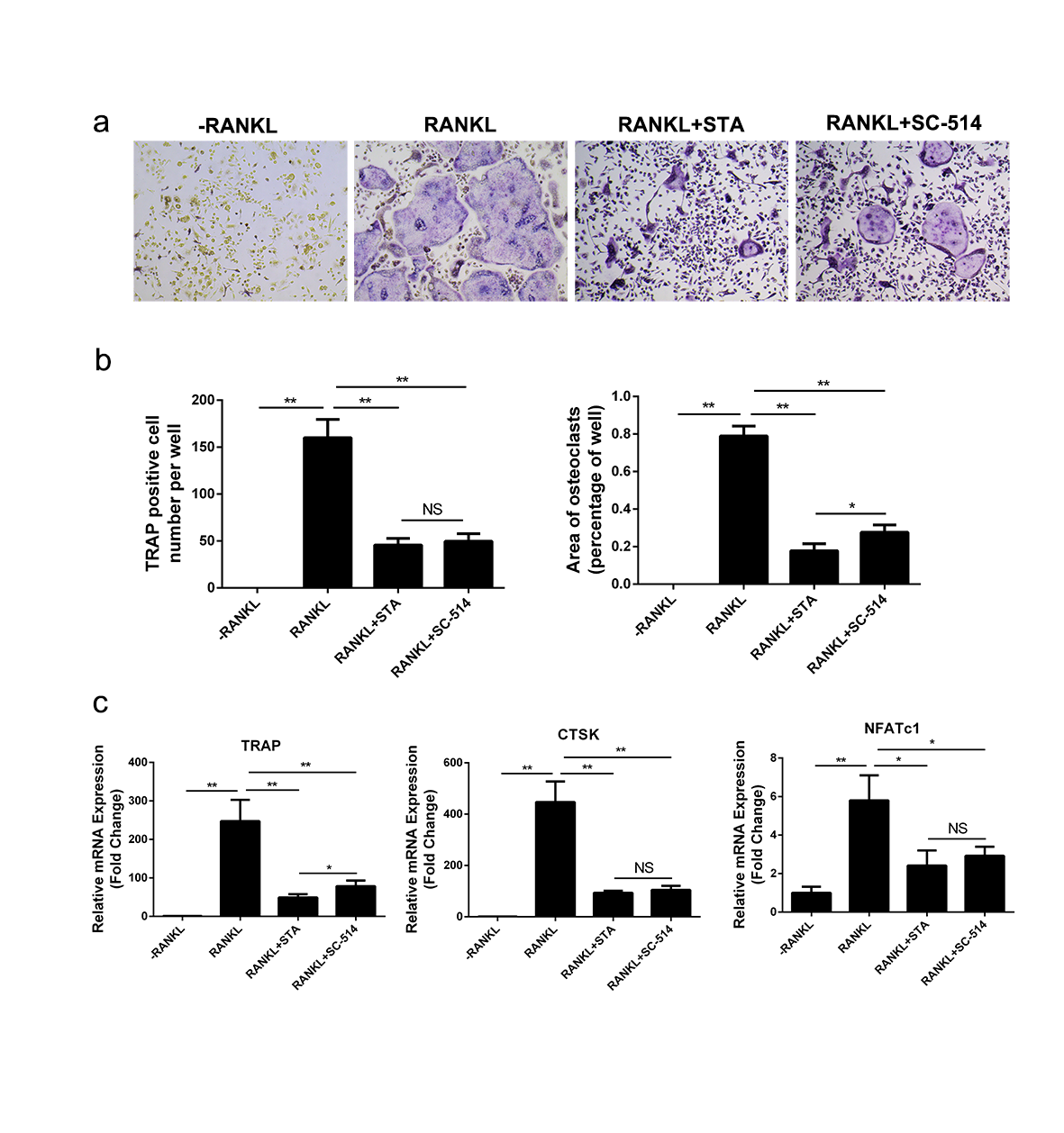

Supplement: Supplementary file 3 [file JCMM-23-6730-s003.tif]

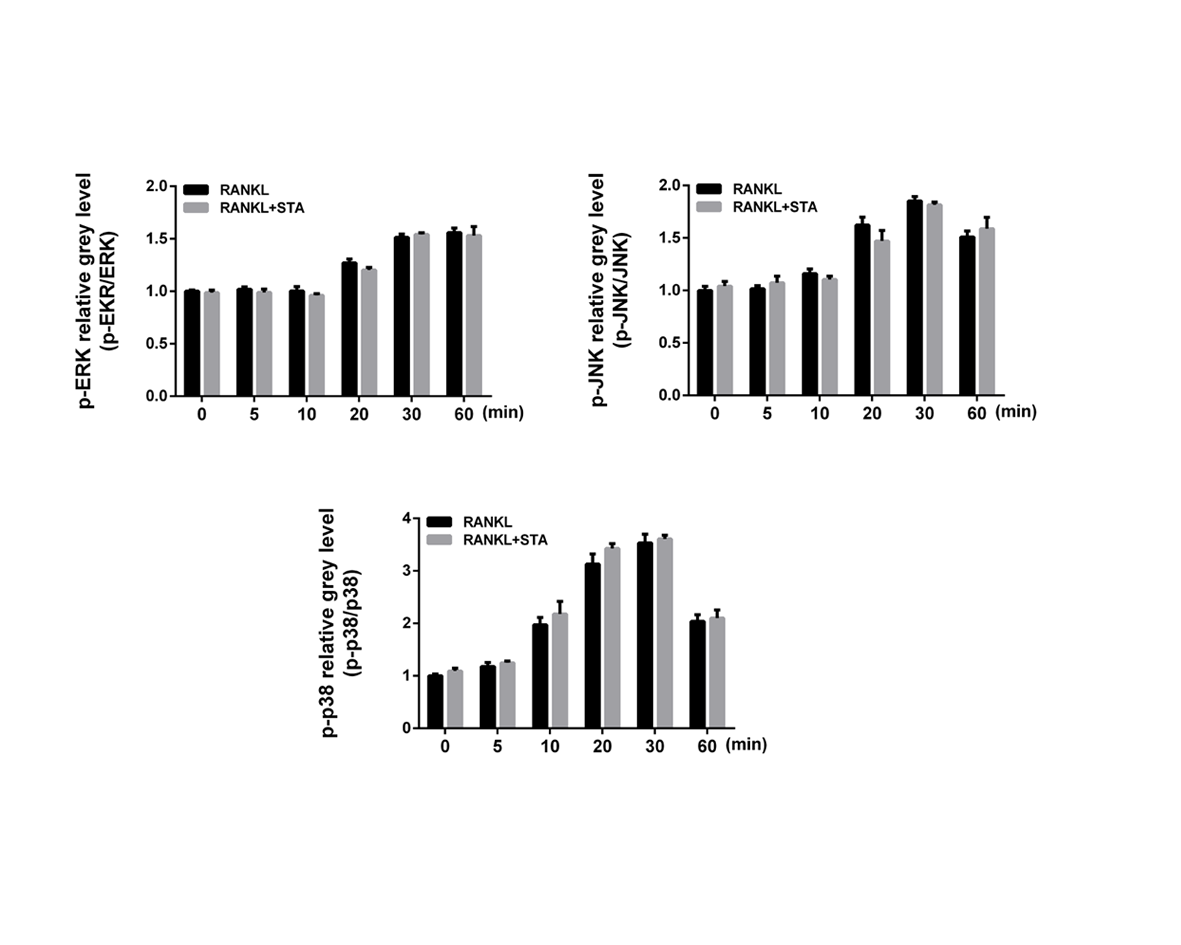

Supplement: Supplementary file 4 [file JCMM-23-6730-s004.tif]
