# Supplementary material for: Stachydrine prevents LPS‐induced bone loss by inhibiting osteoclastogenesis via NF‐κB and Akt signalling
Source: J Cell Mol Med. 2019 Jul 21;23(10):6730–43. doi: 10.1111/jcmm.14551 (PMC6787569; doi:10.1111/jcmm.14551)
Supplement: Supplementary file 5 [file JCMM-23-6730-s005.docx]

**Legends to Supplementary Figures**

Supplementary Figure 1. STA do not affect RANKL-induced TAK1 activation. The gray level of phosphorylated TAK1 were quantified and normalized relative to total TAK1.

Supplementary Figure 2. STA and SC-514 inhibit RANKL-induced activation of NF-κB signaling. (a) BMMs were pretreated with 100 μM STA, 5 μM SC-514 or DMSO for 4 h, followed by stimulation with 50 ng/ml RANKL for 10 min. Whole-cell lysates were analyzed by performing western blotting analysis. (b) The gray levels of phosphorylated p65, and IKKα/β were quantified and normalized relative to their total protein counterparts. The grey levels of p-IκBα and IκBα were normalized to β-tubulin. *P < 0.05, **P < 0.01, NS, not significant, compared with the RANKL alone group.

Supplementary Figure 3. STA and SC-514 inhibit RANKL-induced osteoclast differentiation and osteoclast-related gene expression. (a) BMMs were cultured with 25 ng/ml M-CSF and 50 ng/ml RANKL, in the presence of 100 μM STA, 5 μM SC-514 or DMSO for 5 days. Cells were fixed and TRAP staining was performed. (b) The number and area of TRAP-positive cells were analyzed. (c) The mRNA expression of the osteoclast-related genes TRAP, CTSK and NFATc1 was determined by performing qPCR. *P < 0.05, **P < 0.01, NS, not significant, compared with the RANKL alone group.

Supplementary Figure 4. STA do not affect RANKL-induced MAPK signaling. The gray levels of phosphorylated ERK, JNK and p38 were quantified and normalized relative to their total protein counterparts.
